# Quantification of right ventricular function from short-axis displacement-encoded images

**DOI:** 10.1186/1532-429X-16-S1-P52

**Published:** 2014-01-16

**Authors:** Jonathan D Suever, Gregory J Wehner, Linyuan Jing, David Powell, Christopher M Haggerty, Xiaodong Zhong, Frederick H Epstein, Brandon K Fornwalt

**Affiliations:** 1 Pediatrics, University of Kentucky, Lexington, Kentucky, USA; 2 Biomedical Engineering, University of Kentucky, Lexington, Kentucky, USA; 3 MR R&D Collaborations, Siemens Healthcare, Atlanta, Georgia, USA; 4 Biomedical Engineering, University of Virginia, Charlottesville, Virginia, USA

## Background

Right ventricular (RV) function is important in many disease states, but is difficult to quantify from routine MR imaging. Previous work has shown that long-axis deformation/strain is the most critical contributor to global RV function; however, short-axis datasets allow for better coverage of the RV. Thus it would be ideal to be able to quantify RV long-axis function using short-axis slice orientations. We hypothesized that a stack of three-dimensional (3D) displacement encoded (DENSE) images could reliably quantify longitudinal deformation of the RV to overcome the need for acquiring additional long-axis views of the RV.

## Methods

**Figure 1:**
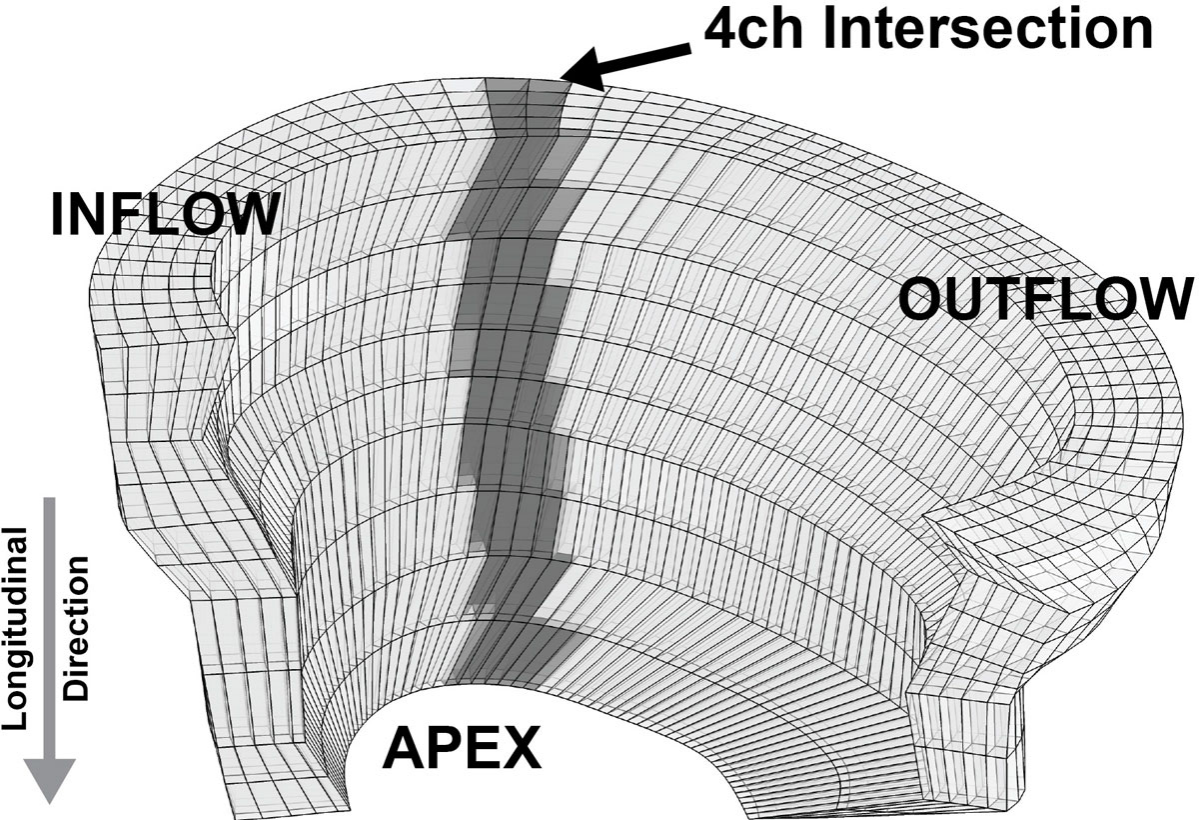
**Three-dimensional reconstruction of the right ventricle from short-axis cine DENSE images**. Longitudinal strains derived from three-dimensional DENSE were compared to strains derived from a standard four-chamber (4 ch) image at the overlapping mesh elements (grey region).

A contiguous stack of cine short-axis DENSE images encompassing the entire RV was acquired with 3D encoding in eight healthy volunteers (Age: 27 ± 3 years) using a 3T Siemens Tim Trio scanner. Endo- and epicardial boundaries were manually drawn on each image to generate a 3D reconstruction of the RV myocardium (Figure 1). The measured displacement field was used to deform the mesh and longitudinal strains were computed at every point throughout the volume. For comparison to the short-axis stack with 3D encoding, a standard four-chamber DENSE image with two-dimensional in-plane displacement encoding was acquired. Similar to the 3D analysis, a mesh was deformed using the measured displacements and was subsequently used to determine longitudinal RV strain values. For comparison with the four-chamber data, only short-axis points lying within the four-chamber imaging slices were used to compute peak longitudinal strain (grey in Figure 1). All strains were compared using a two-tailed paired t-test.

## Results

**Figure 2:**
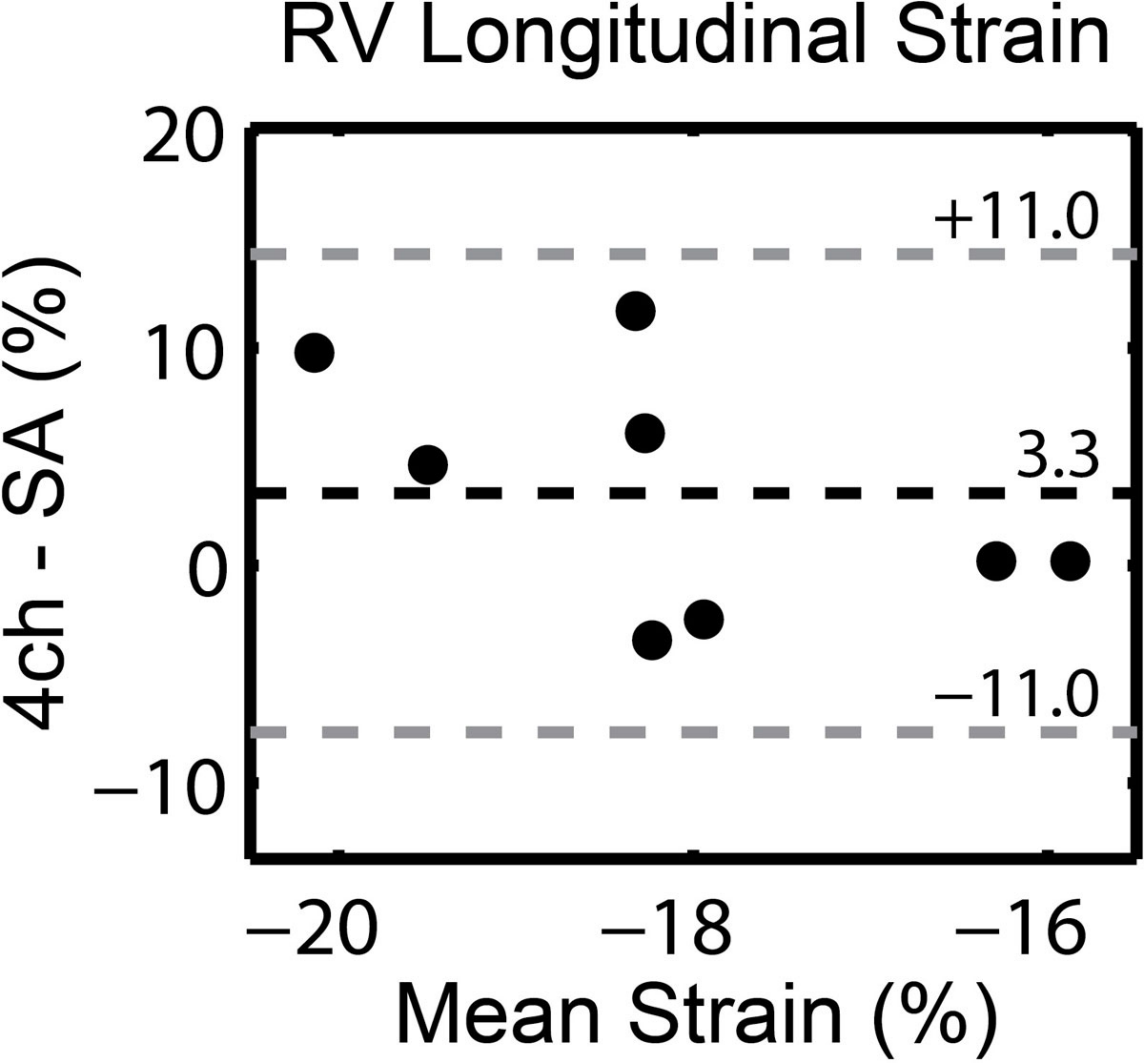
**Bland-Altman analysis revealed a non-significant bias of 3.3% with short-axis (SA) longitudinal strain values being larger than those derived from the four-chamber (4 ch) images**.

Right ventricular longitudinal strains derived from short-axis 3D DENSE images (-20 ± 4%) were comparable to values obtained from four-chamber images (-16 ± 2%) (p = 0.14). In addition to obtaining information solely at the four-chamber/short-axis intersection, we computed a global RV longitudinal strain of -17 ± 2% from 3D DENSE data (p = 0.64 relative to four-chamber only). Bland Altman analysis yielded a non-significant bias of 3 ± 11% between four-chamber and short-axis longitudinal strain estimates (Figure 2).

## Conclusions

We have demonstrated that short-axis 3D DENSE imaging allows for accurate characterization of right ventricular longitudinal strain compared to a standard long-axis four-chamber acquisition which is typically used to look at RV function. In addition, 3D DENSE acquired in a short-axis orientation allows for more complete coverage of the RV compared to acquisitions based on long-axis image planes. It is likely that the more complete assessment of RV function provided by 3D DENSE could potentially improve upon the accuracy, reproducibility and prognostic ability of common echocardiographic techniques such as the tricuspid annular plane systolic excursion (TAPSE), but future work will need to investigate this.

## Funding

This research was funded in part by an NIH Early Independence Award to BKF (DP5 OD012132); contributions made by local businesses and individuals through a partnership between Kentucky Children's Hospital and Children's Miracle network; and the University of Kentucky Cardiovascular Research Center, grant UL1RR033173 from the National Center for Research Resources (NCRR), funded by the Office of the Director, National Institutes of Health (NIH) and supported by the NIH Roadmap for Medical Research. The content is solely the responsibility of the authors and does not necessarily represent the official views of the funding sources.

